# Syrian refugees, between rocky crisis in Syria and hard inaccessibility to healthcare services in Lebanon and Jordan

**DOI:** 10.1186/1752-1505-7-18

**Published:** 2013-09-03

**Authors:** Ziad El-Khatib, David Scales, Jo Vearey, Birger C Forsberg

**Affiliations:** 1 Department of Public Health Sciences, Health Systems and Policy Group Global Health, Karolinska Institute, Tomtebodav. 18a, 171 65 Solna, Sweden; 2 World Health Programme, Université du Québec en Abitibi-Témiscamingue (UQAT), Quebec, Canada; 3 Children’s Hospital Informatics Program, Boston Children’s Hospital, Harvard Medical School, 10 Shattuck St., Boston 02115, MA, USA; 4 African Centre for Migration & Society, University of the Witwatersrand, Johannesburg, South Africa

**Keywords:** Syria, Refugees, Lebanon, Jordan, Access to healthcare

## Abstract

Around 3% of the world’s population (n = 214 million people) has crossed international borders for various reasons. Since March 2011, Syria has been going through state of political crisis and instability resulting in an exodus of Syrians to neighbouring countries. More than 1 million Syrian refugees are residents of Lebanon, Jordan, Turkey, Egypt and North Africa. The international community must step up efforts to support Syrian refugees and their host governments.

## Background

**Figure 1:**
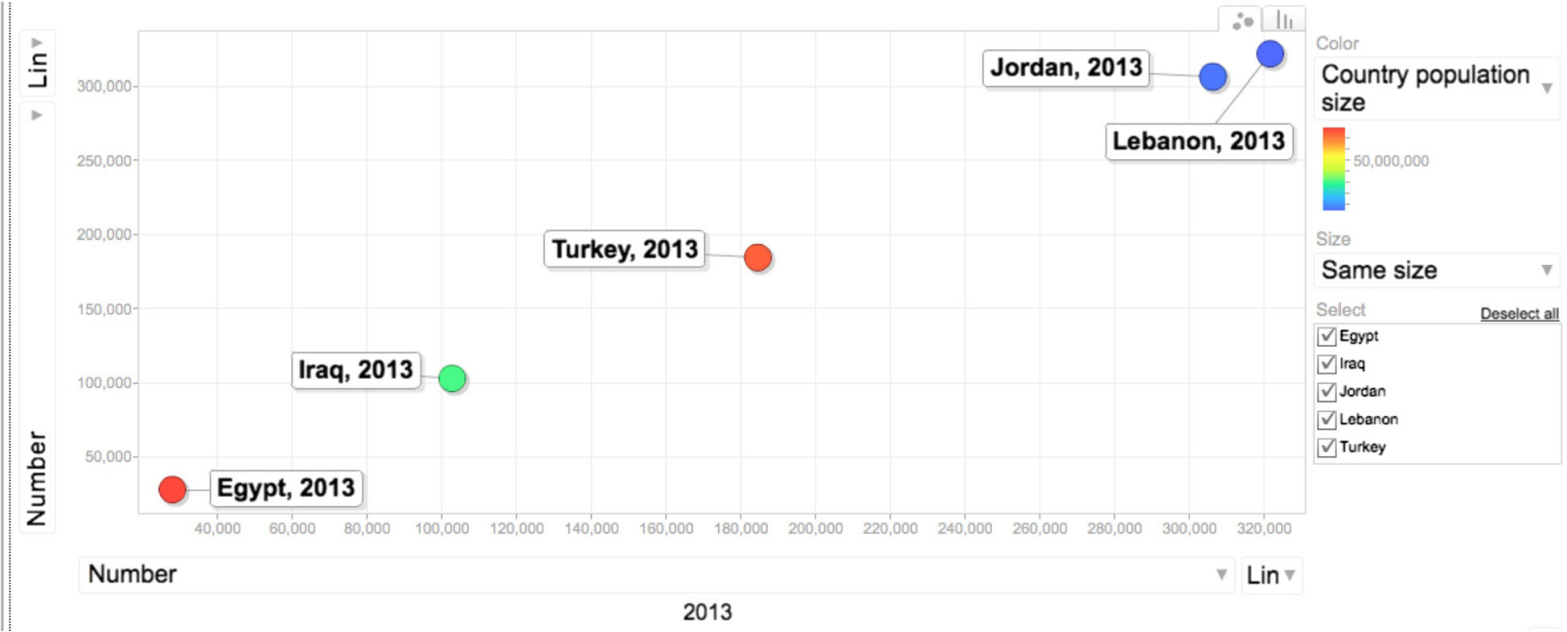
Distribution of Syrian refugees in the Middle East.

Since March 2011, Syria has been going through state of political crisis and instability resulting in an exodus of Syrians to neighbouring countries. By the end of May 2013, The World Health Organization (WHO) estimates over 1.5 million refugees in Lebanon, Jordan, Turkey, Egypt and North Africa [1]. Almost the majority (75%) half are below age 18 (54%) and the plurality (34%) are in Lebanon (Figure 1).

The estimated population of Lebanon is 4.2 million, which makes the Syrian refugees 7% of its population size. Yet, the medical response of the needs to this population has not been met [2].

**Figure 2:**
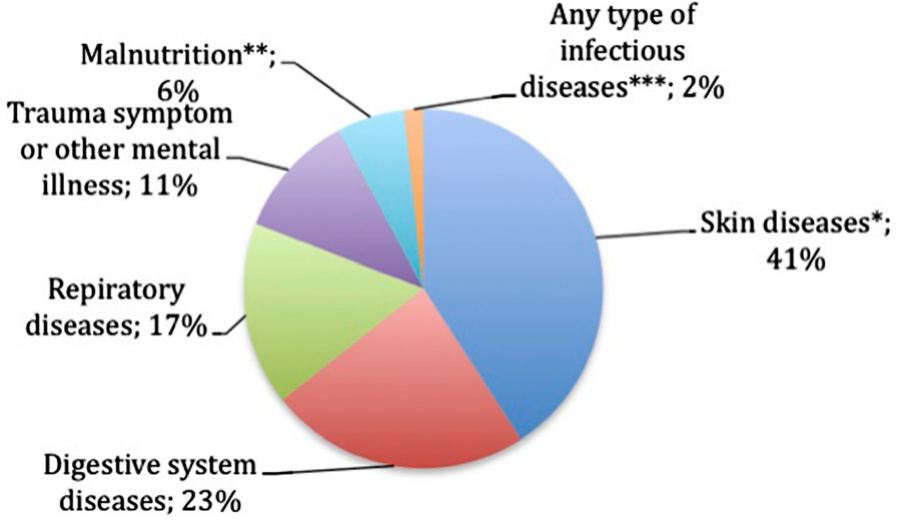
**Distribution, by percentage (%), for the different types of diagnosis among the Syrian patients in Lebanon.** * Skin diseases include leishmaniasis, scabies, lice and staphylococcal skin infection; ** Malnutrition was predominantly among children; *** Infectious diseases include measles, jaundice and typhoid.

The recent Médecins Sans Frontières (MSF) report surveyed the main needs of the refugees in Lebanon. Basic needs like primary health care, treatment of chronic diseases, and ante-natal care [3] (Figure 2) [2] could be easily met in Lebanon thanks to a substantial healthcare infrastructure that includes a total of 165 hospitals and 158 primary healthcare centres [4] distributed over a relatively small geographic area (10,452 km^2^) and the staff in those health services is highly competent.

Only 2% of more than 5,000 drugs in Lebanon are generic [5]; branded medications are highly priced, in addition the refugees must pay out of pocket to access to the Lebanese healthcare system and services are costly [3].

Overall, refugees are the weakest and most vulnerable category in a conflict setting. As long as the crisis continues in Syria, most refugees cannot return and therefore their medical needs are likely to require assistance in the refuge setting. Providing services to them in Lebanon appears to be a matter of both human and political priority. Options for engaging medical services in Lebanon in the care of Syrian refugees in the country should be urgently looked into by the Lebanese government.

In Jordan, the situation is similar. With up to 3,500 Syrians crossing every day, the rate at which refugees have poured over Syria’s southern border has outstripped the ability of the Jordanian government and the international community to ensure adequate access to health services for refugees living inside Za’atari, the main refugee camp, and those dispersed throughout Jordan [6].

Within Za’atari camp, the needs of refugees have already surpassed the camp’s original design, creating sanitation problems as well as making it difficult to access proper medical care [6]. The pace of refugees entering the camp makes it a challenge to provide adequate water and sanitation. Medical care is provided free of charge by various relief services, e.g. French, Moroccan, MSF, Saudi to name only a few. However, the medical care provided prioritizes urgent care needs only. With the crisis stretching over two years, chronic diseases may be overlooked as routine blood tests, e.g. thyroid stimulating hormone, and medications such as asthma inhalers are either not available in the camp, or only intermittently available.

Outside of Za’atari refugees face different challenges. Unregistered refugees, i.e. Syrians that crossed the border either legally or undetected by Jordanian security, must wait weeks to months for their UNHCR interview [7]; they must pay out of pocket for medical services or supplies in the meantime. One Syrian patient with diabetes and a kidney transplant waited over a month for his interview, during which time he was unable to get refills of immunosuppressant medications and insulin^a^. Unfortunately, free services offered by the Jordanian government like vaccinations are not widely known to urban refugees, resulting in Syrian children going unvaccinated, as parents do not seek care if they perceive they must pay out of pocket [7].

Traditionally, health responses to forced migration in times of civil war or natural disaster focus on emergency (humanitarian) needs, and the management of communicable diseases. As the drivers of forced migration shift to on-going political crises and instability, associated health-responses must also change gear in order to engage with different health needs [8]. Contemporary contexts associated with forced migration present different healthcare challenges, reflecting the health profile of the region. Current responses in Lebanon and Jordan struggle to engage with the more prevalent, chronic, non-communicable health needs of refugees and asylum seekers from Syria. This includes access to primary healthcare for chronic disease management and antenatal care; needs that could be met by the existing national healthcare systems of Lebanon and Jordan, countries with strong public healthcare systems.

In May of this year, the MSF reported the increase in number of patients coming from Damascus and residing in the already over-crowded Palestinan refugees camp at Ain el-Helweh, south of Lebanon [9]. Should the Syrian conflict persist and expand in Damascus, there is risk of an additional 1 million refugees fleeing to Lebanon [2]. While host governments and the international community have been responding to meet the needs of Syrian refugees, the unabated tide of emigration from Syria has rendered these responses inadequate. The international community must step up funds to support Syrian refugees and their host governments.

## Endnote

^a^ Personal observations based on the interaction of DS with patients.

## Competing interests

The authors declare that they have no competing interests.

## Authors’ contributions

ZEK contributed to the conception of the idea and first draft of the manuscript. DS contributed to the section of Jordan and the text. JV contributed to the text. BCF contributed to the text through advice and development of the manuscript and gave guidance on correspondence with Lebanese colleagues. All authors read and approved the final manuscript.
